# Learning at large conferences: from the ‘sage on the stage’ to contemporary models of learning

**DOI:** 10.1007/s40037-017-0351-3

**Published:** 2017-04-11

**Authors:** Dario Torre, Annalisa Manca, Steven Durning, Janusz Janczukowicz, David Taylor, Jennifer Cleland

**Affiliations:** 1 Graduate Program in Health Profession Education, Uniformed Services University Of Health Sciences, Bethesda, MD USA; 2 School of Medicine, Medical Education Institute, University of Dundee, Dundee, UK; 3 Centre for Medical Education, Medical University of Lodz, Lodz, Poland; 4 School of Medicine, Institute of Learning and Teaching, Faculty of Health and Life Sciences, University of Liverpool, Liverpool, UK; 5 Institute of Education for Medical and Dental Sciences, University of Aberdeen, Aberdeen, UK

**Keywords:** Flipped classroom, Conference, Innovation

## Abstract

**Aim:**

To explore and evaluate the affordances of a flipped classroom model applied to a research paper session within the professional development opportunity of a large conference setting.

**Method:**

Authors were invited to present their research papers in a flipped classroom presentation format at two large, multi-national conferences. Before the session, authors and moderators met online to clarify features of the session, and preparation of the material. The research material was then posted online before the conference, to allow access by meeting attendees. During the sessions, moderators encouraged the audience to actively participate. An evaluation form was collected from the audience at the end of each session.

**Results:**

Participants found the session valuable, and appreciated the opportunity to engage in a meaningful dialogue with colleagues. However, the majority of the audience did not access the materials in advance. Lack of time, or technology-related issues were mentioned as potential challenges to such format.

**Conclusion:**

In the context of a large conference, a ‘flipped session’ format can facilitate active learning and a participatory culture of inquiry. However, to change the nature of how individuals learn collaboratively at large conferences means a change in the culture of continuous professional learning.

## Introduction

Although we strive for contemporary and creative approaches to teaching and learning in medical education, the formats used at professional development conferences remain mostly of the ‘sage on stage’ [1] variety. Yet there are other, more contemporary formats to meet the professional development needs of educators which may be suitable for such global and cross-cultural contexts as a large international conference [1]. These include ‘Unconferences’, organized around participant-driven discussions on topics of interest, which replace traditional keynotes or sessions, and/or post-plenary discussions. Hackathons [2] and BarCamp [3] are open events in which people from different disciplines and with different skillsets collaboratively work to develop innovative ideas and find creative technology-based solutions to key challenges. Similarly, massive open online courses (MOOC) and webinars can offer the opportunity to present material in advance of face-to-face meetings [4].

In this paper we focus on a specific format: flipped learning [5–7]. The flipped classroom, which often employs easy-to-use technology to deliver content to learners outside the classroom, allows valuable face-to-face time to be used for group discussion and interaction, to (co)construct knowledge and problem-solving activities [8, 9]. While the (co)constructive and participatory approach of the flipped classroom has much appeal for professional development contexts, a literature search did not identify any studies reporting the use of the flipped classroom in a conference setting. As a consequence of this gap in the literature, we do not know how these models are embraced by educators for their own professional learning and development. This is important as educator views towards, and engagement with, contemporary models of teaching and learning may have implications for how these are adopted in everyday medical education practice.

In this paper, we describe and evaluate the use of a flipped classroom model in the context of a large, multi-national conference, the Association for Medical Education Europe (AMEE) 2014 and 2015 scientific meetings. Our experiences and feedback from participants add to the conversation about using participatory models for professional development specifically, and their use for teaching and learning generally.

## Methods

### Pre-session

The AMEE research committee sent an email message to all authors who had their research papers accepted at the AMEE 2014 and 2015 meetings, inviting them to consider presenting their research in a flipped classroom presentation format. The message also contained a brief explanation of the session and a presentation illustrating the main concepts of the flipped classroom approach, and how it would be applied in the conference setting. Ten authors, from the USA and Europe, responded positively to this email, allowing us to fully populate each flipped classroom session (one per meeting, five presenters per session).

Several months before each meeting, a conference call was held with the presenters and a number of the facilitators, to discuss and clarify aspects of the session. Authors were given guidance on what they needed to prepare in advance; the overarching guidance to authors was to create the material in the way they felt would be most useful for the audience given the nature of their research. We suggested emphasizing specific aspects of their research to encourage participation and discussion, such as: methodological dilemmas, theoretical frameworks, future research questions.

Authors submitted this material in advance for review by members of the AMEE Research Committee. The aim of this review was to identify potential technical issues or other barriers to access in addition to allow presenters to recognize and emphasize larger themes that cut across the multiple presentations. Peer-review and online discussion were encouraged among authors, to model and embed the participatory and (co)constructive aims of this session. After this process, finalized materials were posted on the MedEdWorld website 6–8 weeks before the conference, to allow delegates to access the material and become familiar with the format and goals of the session in advance of the conference.

### The research papers ‘flipped classroom’ style sessions

Each session included five authors, two moderators and conference attendees who had self-selected to attend these sessions. The moderators were members of the AMEE Research Committee with prior experience of using the flipped classroom model in other contexts, who had been involved in the pre-session activities. Each author was given two minutes to introduce themselves and state the title and purpose of their research paper, without any visual aids. The remaining 80 min of the session were used for discussion. The moderators encouraged the audience to actively participate, ask questions and seek clarification about different aspects of the research, as well as to reflect on issues and ideas emerging from the group discussion.

### Evaluation

A brief paper-based evaluation form was given to the audience at the end of each session. The evaluation form consisted of five questions (interval, categorical and open-ended) inviting them to rate: the overall educational value of the session; whether they had reviewed the material in advance; and whether they would be interested in having more flipped classroom sessions in future meetings. We also asked what they found most interesting about the session, and if they had any suggestions for improvement.

We analyzed this data using descriptive statistics on interval and categorical questions, and a thematic analysis [10] of the open-ended responses.

## Results

We collected 47 evaluations from approximately 25 participants per session. Of the participants, 88% rated the session highly (22 very good/excellent, 20 good). The majority of the participants (75%, *n* = 35) had not reviewed the preparation material. The most common reasons for this were: lack of time, not being clear about the need to do so, or technical inability to access the material. A total of 79% (*n* = 37) reported that they would be interested in having more ‘flipped sessions’ in future meetings.

Participants reported that the most interesting feature of the session was the opportunity to engage in an in-depth and interactive discussion with fellow audience members and with the authors. Suggestions for the future included organizing the session around topics with a common theme and enabling online engagement in advance of the session.

## Discussion

To the best of our knowledge, this is the first report of the use of a flipped classroom model at an international professional development conference. This approach was favourably evaluated by the audience. However, there were a number of lessons learned and observations which will be useful for those considering using this approach in similar contexts in the future.

First, and in accordance with the wider literature [8, 9, 11], the preparatory stage is time intensive. Early communication with authors, support and advice with the design process are necessary, particularly if authors have minimal experience with the flipped model. We found that preparation is worthwhile not just for information sharing but also in terms of developing a culture of participation between the authors/presenters and the moderators, which contributes to the smooth running of the session.

However, we also identified that most of the audience did not access any of the materials in advance. This is a key issue, as a flipped model, or indeed any approach to preparatory online learning, cannot support positive learning experiences if attendees do not engage with the material in advance of the face-to-face discussion time [11]. This lack of engagement did not seem due to the nature of the advance materials (e. g., stimulating or not) but rather seemed associated with factors which have implications for bringing innovative formats to a large, internationally diverse conference.

The first of these was technological issues. In the setting of a large meeting attended by educators who have a wide, often unknown, range of technology skills and infrastructures, the use of technology may constitute a barrier to the implementation of a flipped model. Familiarity with technology, usability, accessibility to all delegates and potential technical problems have to be balanced with the benefits of using multimedia online material. Thus it is important to consider possible technological impediments when planning a flipped presentation session that involves a worldwide audience. Session organizers should try and anticipate some of the technological barriers that may present across different systems and countries by piloting access to material from different platforms and computer systems.

Second, even with pre-conference advertising, audience participants were not completely aware that they were expected to prepare in advance. Better advertising of the flipped session and its format and an email with a link to the presentation material (or a Facebook/Twitter alert) sent in advance may help in this respect. Putting a specific call for flipped classroom papers on predefined topics with all conference advertising, particularly the calls for submissions, may also help. Having common themes among research papers within a session will enable authors to prepare questions for each other and will facilitate interaction with the audience, while furthering the construction of meaning through social interaction and inquiry – although this may be difficult to manage in a conference situation where papers tend to be loosely grouped.

However, and coming to our third point, the issue of non-engagement may be less to do with technological barriers or advertising and more to do with people’s perception of their role as conference participants. Anecdotally, it seemed that those in the audience did not plan on attending the flipped session in advance and did not realize the nature of this new session. This may be due to the influences of an outdated, yet still common, approach to professional learning and development in which a ‘sage on the stage’ [5] transmits knowledge to learners, who are seen as passive recipients of this knowledge rather than engaged and active learners who (co)construct knowledge in.

Yet, with the advent of generational and technological changes, expectations of how to participate at personal development events are changing. There are more examples of personal development activities in the setting of professional association meetings which involve elements of active [12] and cooperative learning [13], such as engagement in the learning process, promotive interaction, creative thinking, group processing, and collective participation. All of these factors contribute to create a professional culture leading to a common understanding of problems, methods and solutions [14] in global, cross-cultural contexts to respond to the needs of educators worldwide.

## Conclusion

In conclusion, we believe that, within any professional development conference, a ‘flipped session’ could foster reflection and active learning, support collaborative construction of meaning and understanding through discourse, and create a learning environment where a participatory culture of inquiry may even carry beyond the time of the meeting. However, to change the nature of how individuals connect with each other at any large conference means a change in the culture of professional learning in this kind of setting. The use of new educational and social learning formats at large conferences requires further research and evaluation.
